# Secret Terms in Physicians' Prescriptions

**Published:** 1916-03-18

**Authors:** 


### The Hospital

The Workers' Newspaper of Administrative Medicine and Institutional Life, Administration, National Insurance and Health.

No. 1553, Vol. LIX. SATURDAY, MARCH 18, 1916. PRICE ONE PENNY.

### SECRET TERMS IN PHYSICIANS' PRESCRIPTIONS.

In essence the physician's prescription is a written direction for the preparation or compounding of a medicine. What substances shall enter into the prescription, and in what doses they shall be administered, are questions on which the writer is free to exercise his individual judgment and choice, it being, of course, understood that he accepts responsibility for his decisions and orders. There is only one qualification of this position—namely, that if the prescription contains any name which is mentioned and defined in the British Pharmacopœia, this name must be used in the pharmacopœial sense by the physician and interpreted in the pharmacopœial sense by the dispenser. Outside this one qualification the physician is free to frame his prescription at his own will and in any terms he may deem suitable. Hence, as a matter of strict right, he can, if he is so disposed, use a term or terms of which the meaning is known only to himself and to some selected individual pharmacist. There is no duty incumbent on the physician to employ terms to which all pharmacists hold the key, nor is he under any obligation to leave the dispensing of the prescription to any pharmacist whom the patient may care to select. It is quite within his choice both to select the dispenser and to write his directions in a secret code known only to this dispenser and himself. There are, we understand, simple souls who will be surprised at this statement and who believe that the physician is limited both in the selection of remedies and in the terms in which he describes them by some mysterious authority whose power is over all. But this is a pure delusion, and the true position is as we have just stated it.

Nevertheless, whatever be the rights of the matter, the custom of the profession undoubtedly is to use in prescriptions terms which have an authoritative interpretation and which are recognised and understood by all qualified pharmacists. The first reason for such a practice is the convenience of the patient. Obviously this is promoted by an arrangement which makes the dispensing of the prescription a possibility in all parts of the kingdom as compared with a plan which would confine such action to a single locality and establishment. Further, it must be admitted that public opinion favours the view that the prescription is purchased by the fee, that it is therefore the property of the patient, and that he has the right to have it dispensed as often as pleases him, whether for his own benefit or for the benefit of his confiding friends. Whether such a pretension is sound in law or in morals may be doubted, but it exists, and anything like an organised attempt to defeat it by the use of a private code would probably excite much opposition. Another explanation of the existing custom is the natural dislike of anything which savours of secrecy and mystery in the practice of medicine, and this influence is in the profession a growing rather than a decreasing quantity. The prophet who will " strike his hand over the place and recover the leper " has no doubt been prominent in certain phases of medical history, and perhaps even yet is not wholly extinct. But his glory has for the most part disappeared, and both doctors and the lay public are content to recognise that medical practice rests on the homely basis of commonsense reinforced by technical knowledge. Last of all, among the objections to secret terms in prescriptions is the fact that such a custom argues the existence of some private arrangement between the prescriber and the favoured dispenser, with the almost inevitable suspicion that at the back of the proceeding there is some financial advantage to the physician. Anxious to avoid even the appearance of evil, the profession does not look with favour on any proceeding which might be regarded as affording cover for the passing of a business commission, and one evidence of this attitude is the general cultivation in prescriptions of terms which are open to the interpretation of every pharmacist. At the same time, we repeat, there is nothing illegal or immoral in the opposite custom, and there may be occasions in which a physician feels himself justified in adopting it.

This question of private terms in prescriptions has recently been raised in connection with the National Insurance Act, and here the custom has been authoritatively condemned. But the condemnation does not rest on any broad claim that the practice is inherently vicious, but only on the contention that in the special circumstances under consideration it is inadvisable and unfair. The insured person, in a certain restricted and limited sense, is understood to enjoy "free choice of doctor," which is found in practice to mean that, provided due notice is given, he can change his medical adviser once every twelve months. But in the fullest sense of the term he has "free choice of pharmacist," for he is at liberty to take his doctor's prescription to, and obtain his medicines from, any one of the pharmacists whose names are entered on the Insurance panel. Obviously this is fair both to the insured person and to the pharmacists, and there could be no advantage in interfering with the liberty thus accorded. But if a medical practitioner deliberately phrases his prescription, not in the usual language of prescriptions but in terms and symbols of which the meaning is unknown to the general pharmaceutical world, the liberty of the patient obviously will be curtailed. He must needs carry his prescription to some pharmacist who holds the key of the mystery, and who in his turn has been educated to this level by the writer of the prescription. In a word, the free choice of pharmacist granted to the assured by Act of Parliament will be destroyed by some secret arrangement made between the writer of the prescription and some individual dispenser. It is sufficient to state this position to secure its condemnation, and hence we are in entire sympathy with the decision which affirms that prescriptions written under the National Insurance Act must be in terms which are open to the confident interpretation of every pharmacist. To admit this, however, does not in the least compromise the freedom of the physician outside the range of this Act, and the fact that the question has been raised in this limited sphere makes it advisable to state this freedom in terms free from all ambiguity.

